# Hypothesis of Long-Term Outcome after Coronary Revascularization in Japanese Patients Compared to Multiethnic Groups in the US

**DOI:** 10.1371/journal.pone.0128252

**Published:** 2015-05-29

**Authors:** Taku Inohara, Shun Kohsaka, Masashi Goto, Yutaka Furukawa, Masanori Fukushima, Ryuzo Sakata, MacArthur Elayda, James M. Wilson, Takeshi Kimura

**Affiliations:** 1 Department of Cardiology, Keio University School of Medicine, Tokyo, Japan; 2 Kyoto University Health Service, Kyoto, Japan; 3 Division of Cardiology, Kobe City Medical Center General Hospital, Kobe, Japan; 4 Translational Research Center, Kyoto University Hospital, Kyoto, Japan; 5 Department of Cardiovascular Surgery, Kyoto University Graduate School of Medicine, Kyoto, Japan; 6 Division of Biostatistics, Texas Heart Institute at Baylor St. Luke’s Medical Center, Houston, Texas, United States of America; 7 Division of Cardiology, Texas Heart Institute at Baylor St. Luke’s Medical Center, Houston, Texas, United States of America; 8 Department of Cardiovascular Medicine, Kyoto University Graduate School of Medicine, Kyoto, Japan; Innsbruck Medical University, AUSTRIA

## Abstract

**Background:**

Ethnicity has a significant impact on coronary artery disease (CAD). This study investigated the long-term outcomes of Japanese patients undergoing revascularization compared with US patients belonging to multiple ethnic groups.

**Methods and Results:**

We evaluated clinical outcomes, based on ethnicity, of patients included in the Coronary Revascularization Demonstrating Outcome (CREDO-Kyoto) and the Texas (US) Heart Institute Research Database (THIRDBase) registries. For the analysis, we included 8871 patients from the CREDO-Kyoto registry (median follow-up period [FU], 3.5 years; interquartile range [IQR], 2.6–4.3) and 6717 patients from the THIRDBase registry (FU, 5.2 years; IQR, 3.8–6.5) who underwent percutaneous coronary intervention or bypass surgery. Cox proportional hazard models were constructed to compare the adjusted long-term outcomes for each ethnic group. A total of 8871 Japanese, 5170 Caucasians, 648 African-Americans, 817 Hispanics, and 82 Asian-Americans were identified. When adjusted, Japanese patients had significantly better outcomes than US patients, classified by ethnicity (Caucasians: hazard ratio [HR], 1.56; 95% confidence interval [CI], 1.35–1.79; Hispanics: HR, 1.53; 95% CI, 1.22–1.93; African-Americans: HR, 2.03; 95% CI, 1.62–2.56), except for Asian-Americans (HR, 0.84; 95% CI. 0.38–1.89) who had outcomes similar to Japanese patients.

**Conclusion:**

Our findings indicate better survival outcomes in re-vascularized Japanese CAD patients compared to major ethnic groups in the US, including Caucasian, Hispanic, and African-American CAD patients. The characteristics and outcomes of Japanese CAD patients were similar to those of Asian-Americans, despite the sample size limitations in the US dataset.

## Introduction

Coronary artery disease (CAD) is one of the leading causes of death in both Eastern and Western countries (e.g., Japan and the US), and a large number of revascularization procedures, including percutaneous coronary interventions (PCIs) and coronary artery bypass grafts (CABGs) are performed.[1–4] However, the lifestyles of the patients in the two regions differ greatly, and little is known about post-revascularization outcomes of Japanese CAD patients or about how these patients compare with their counterparts in the US.

Traditional risk factors, such as age and the presence of comorbid conditions (e.g., diabetes or renal insufficiency), are considered to be predictive of long-term outcomes in both countries. Notably, race and ethnicity are known to greatly influence CAD outcomes,[5, 6] and individuals undergoing coronary revascularization.[7, 8] Previously, a trend towards fewer cardiovascular events in stable and ambulatory CAD patients has been reported for East Asians compared with Western populations.[8] Although previous studies have accounted for ethnic differences,[9–11] most have focused on Caucasians, Hispanics, and African-Americans. Furthermore, these studies were conducted in individual countries and without international comparisons. Therefore, how the demographics and outcomes of Asian patients compare with those of other ethnic groups in the US remains unclear. Given the current situation, it will be difficult to interpret the clinical trials or studies performed in Western countries. Including East Asians along with the various ethnic groups in Western countries in these studies will help determine whether the results are applicable to East Asian countries.

To partially fill this void, we assessed the clinical characteristics and long-term outcomes between Japanese and US patients, from different ethnic groups, using a comparison of information from 2 large databases of patients undergoing (PCIs) and (CABGs) in these countries. The present study structure enabled unique opportunity to describe the long-term outcome of Japanese patients undergoing revascularization strategy in comparison to multiple ethnic groups in the US, and to evaluate which of the US ethnical group would be closest to Japanese patients.

## Methods

The study was conducted in accordance with the Declaration of Helsinki. For THIRDBase, the current study was approved by the institutional review board (Institutional Review Board- Texas Health Resource). Written informed consent was obtained from all enrollees at the time of hospital admission, and all analyzed data were stripped of personal identifiers. For CREDO-Kyoto, Kyoto Univeristy Hosptial Institutional Review Board approved the research protocol. Due to the retrospective nature of enrollment, written informed consent was not obtained from the patients; however, when later contacted for follow-up evaluation, 73 patients were excluded from the analysis because of their refusal to participate in the study. This process is concordant with the guidelines for epidemiologic studies issued by the Ministry of Health, Labor, and Welfare of Japan.

**Fig 1:**
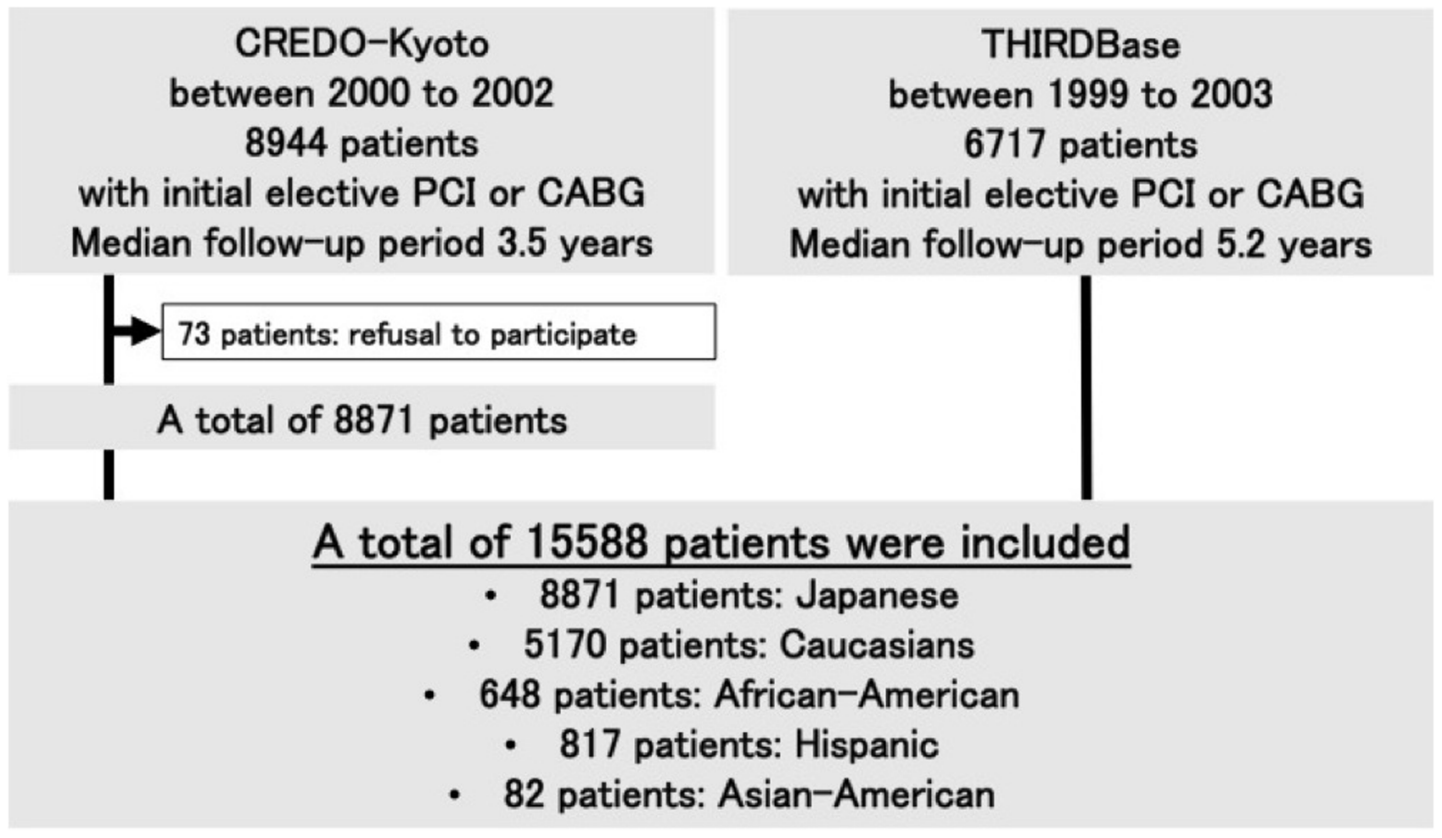
Study Cohort Creation.

We evaluated clinical characteristics and outcomes, based on ethnicity, using the Coronary Revascularization Demonstrating Outcome (CREDO-Kyoto) registry in Kyoto and the Texas Heart Institute Research Database (THIRDBase). CREDO-Kyoto was a multicenter (n = 29) registry based on the network of one tertiary academic hospital (Kyoto University hospital) and its affiliated urban academic hospitals, and THIRDBase is an ongoing single tertiary center registry maintained at the Texas Heart Institute, in Houston, TX, USA. Designed to evaluate the associated periprocedural and late events, both registries, prospectively registered consecutive patients who underwent elective revascularization by either PCI or CABG. Details concerning the designs of CREDO-Kyoto and THIRDBase have been previously reported.[8, 12] CREDO-Kyoto enrolled patients between 2000 and 2002, whereas THIRDBase is a continuous, on-going registry since 1993. For the current analysis, we included THIRDBase patients enrolled between 1999 and 2003 to match the total number of patients in the CREDO-Kyoto registry. The series comprised 15,588 patients, including 8871 patients from the CREDO-Kyoto registry (median follow-up period, 3.5 years; interquartile range [IQR], 2.6–4.3) and 6717 patients from the THIRDBase registry (median follow-up period, 5.2 years; IQR, 3.8–6.5). In order to focus on patients who had undergone initial, elective, isolated revascularization procedures, those who had undergone a previous PCI or CABG operation, required concomitant valve surgery or peripheral vascular revascularization, or were undergoing primary PCI for an acute myocardial infarction (MI), were excluded (Fig 1).

Since drug-eluting stents were not available for use during the study period, all PCI patients underwent placement of a bare-metal stent. Patient histories were obtained through an interview when the patient arrived at the hospital or clinic, and the details were entered into the database. The following variables were documented: left ventricular ejection fraction, number of diseased vessels, urgency of the revascularization procedure, presence of hypertension (characterized by a blood pressure of >130/90 mmHg for THIRDBase and >140/90 for CREDO) or current use of antihypertensive medication, angina severity (Canadian Cardiovascular Society classification), congestive heart failure severity (New York Heart Association [NYHA] functional status), family history of CAD, previous MI, renal function, need for hemodialysis, and presence of peripheral vascular disease (occlusive or aneurysmal vascular disease in the aorta or other peripheral vessels). Coronary anatomic and procedural characteristics, in-hospital outcomes, and vital status as of December 31, 2006, were assessed for all patients.

In the THIRDBase registry, survivorship was determined from the US Department of Vital Statistics Database. For CREDO-Kyoto, follow-up data were obtained from hospital charts or by contacting patients or referring physicians. Both registries had remarkably high follow-up completion rates (THIRDBase, 100%; CREDO-Kyoto, 98% at 1 year and 95% at 2 years). Survival analyses included both in-hospital and long-term survival data; these survival rates were not considered separately.

For the present study, we analyzed the patients in each registry according to their ethnicity. To compare the two registries, we assessed patient demographic characteristics using Pearson’s chi-square test for discrete variables and Student’s *t*-test for continuous variables. We then used the Kaplan-Meier method to draw the survival curve and the log-rank test to identify significant differences in unadjusted survival rates for each ethnicity.

Multivariable Cox proportional hazards models were constructed to control possible confounding factors affecting the long-term cardiovascular outcomes, according to each ethnicity. Age ≥65, gender, obesity, history of myocardial infarction, diabetes, heart failure, NYHA functional class, peripheral vascular disease, renal function, hypertension, hyperlipidemia, family history of CAD, smoking, the number of diseased vessels, and ethnicity were included as a covariate in the multivariate analysis. Because the proportional hazards assumption did not hold for the mode of coronary revascularization, we applied a stratified Cox proportional hazards model using the selected revascularization procedure as the stratification variable. Thus, the variables included in the multivariable model were age ≥65, sex, obesity, history of MI, diabetes, heart failure, NYHA functional class, peripheral vascular disease, renal function, hypertension, hyperlipidemia, family history of CAD, smoking, number of diseased vessels, and ethnicity (Japanese as a reference). Renal function was categorized using a serum creatinine level of ≤179 μmol/L, a serum creatinine level of >179 μmol/L without hemodialysis, and a serum creatinine level of >179 μmol/L with hemodialysis. Cerebrovascular disease was excluded from the multivariable analyses because of the considerable differences in the definitions used by the registries. To assess the validity of the proportional hazard assumption, we plotted-log [-log (survival)] curves for each category of a nominal or ordinal covariate versus log (analysis time).

## Results

The series included 15,588 patients; 8871 patients from the CREDO-Kyoto registry (median follow-up period, 3.5 years; IQR, 2.6–4.3) and 6717 patients from the THIRDBase registry (median follow-up period, 5.2 years; IQR, 3.8–6.5). Table 1 summarizes the patient baseline characteristics, by ethnicity. Overall, the Japanese patients were older and were more likely to be smokers and to have diabetes mellitus. The US patients were more obese and had a greater body mass index. In general, the US patients had a greater prevalence of MI, renal insufficiency, hypertension, dyslipidemia, and family histories of CAD. Among the US patients, African-Americans tended to have more comorbidities than the other ethnicities. Multi-vessel disease was seen more frequently in the Japanese patients than in the US patients, and a similar tendency was recognized among Asian-American patients.

**Table 1:** Baseline characteristics of each ethnic population.

	Total		Japanese		Caucasians		African American		Hispanic		Asian American		
	N = 15,588		N = 8,871		N = 5,170		N = 648		N = 817		N = 82		
	N	%	N	%	N	%	N	%	N	%	N	%	P value
Age (>65 years)	8,603	55.19	5,694	64.19	2,367	45.78	211	32.56	298	36.47	33	40.24	<0.001
Gender (Female)	4,621	29.64	2,585	29.14	1,474	28.51	301	46.45	243	29.74	18	21.95	<0.001
Obesity (BMI <25 kg/m2)	7,795	51.36	2,747	31.41	3,835	77.49	524	82.91	651	84	38	50	<0.001
Previous MI	4,618	29.76	2,327	26.28	1,708	33.34	263	40.78	302	37.15	18	21.95	<0.001
Previous HF	2,326	15.01	1,364	15.43	685	13.37	139	21.55	134	16.48	4	4.88	<0.001
NYHA IV	617	3.98	126	1.43	353	6.9	64	9.92	70	8.61	4	4.88	<0.001
Peripheral vascular disease	1,999	12.88	1,000	11.28	772	15.08	113	17.52	108	13.28	6	7.32	<0.001
Renal insufficiency	1,327	8.61	555	6.35	547	10.68	101	15.66	112	13.78	12	14.63	<0.001
Hypertension	11,122	71.63	6,158	69.46	3,742	73.06	563	87.29	590	72.66	69	84.15	<0.001
Diabetes mellitus	5,540	35.69	3,473	39.2	1,356	26.47	284	44.03	402	49.45	25	30.49	<0.001
Dyslipidemia	8,630	55.63	4,540	51.29	3,202	62.53	355	55.12	483	59.41	50	60.98	<0.001
Family history of CAD	3,575	23.74	1,321	15.72	1,790	34.95	205	31.78	238	29.27	21	25.61	<0.001
Smoking	7,696	50.1	4,608	52.93	2,440	47.69	314	48.68	320	39.41	14	17.07	<0.001
No of diseased vessels													
1-vessel disease	6,023	41.06	3,087	35.13	2,310	50.97	269	48.04	330	46.15	27	36.49	
2-vessel disease	4,719	32.17	2,826	32.16	1,465	32.33	170	30.36	231	32.31	27	36.49	<0.001
3-vessel disease	3,927	26.77	2,875	32.72	757	16.7	121	21.61	154	21.54	20	27.03	

Abbreviations: BMI, body mass index; MI, myocardial infarction; HF, heart failure; NYHA, New York Heart Association; CAD, coronary artery disease.

**Fig 2:**
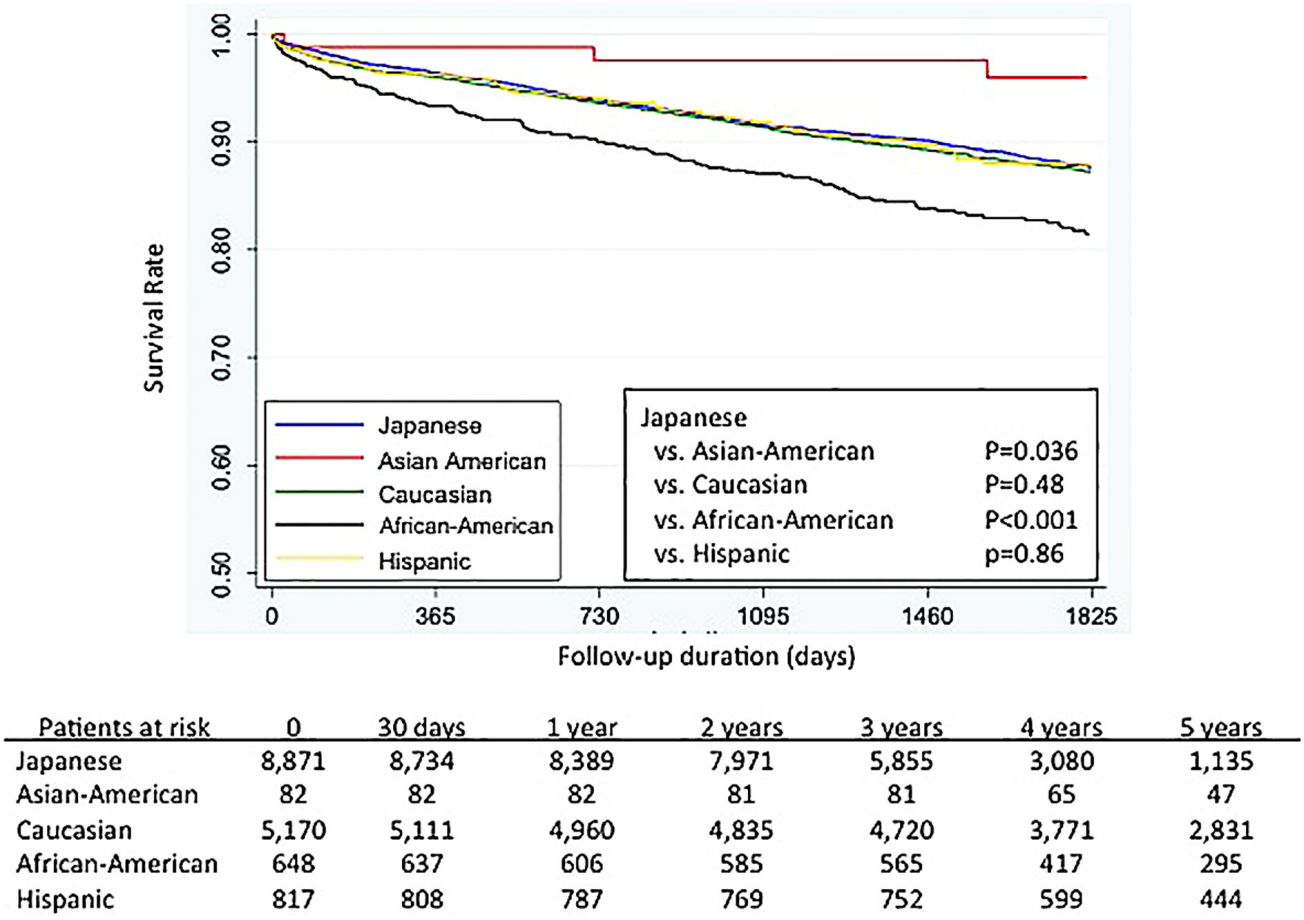
Kaplan-Meyer survival curves, based on patient ethnicity.

Fig 2 shows the Kaplan-Meier survival curves for each major ethnic group (Japanese, Caucasians, Hispanics, African-Americans, and Asian-Americans). The long-term outcomes for Caucasian and Hispanic patients were similar to those for the Japanese patients (Japanese vs. Caucasian p = 0.48, and Japanese vs. Hispanics p = 0.86), whereas the significantly different outcomes were recognized between the Japanese patients and both African-Americans (Japanese vs. African-Americans, p < 0.001) and Asian-Americans (Japanese vs. Asian-Americans, p = 0.036).

After adjustment, Cox-proportional Hazard Models identified age (>65 years), body mass index (>25 kg/m^2^), a history of MI or heart failure, peripheral vascular disease, renal insufficiency, hypertension, diabetes mellitus, hyperlipidemia, and a family history of CAD as significant predictors of mortality (Table 2). Multi-vessel disease was an independent predictor of mortality.

**Table 2:** Results of Cox regression analysis.

	HR (95% CI)	*P* value
Age ≥65 y	2.52 (2.23–2.84)	<0.001
Women	1.05 (0.93–1.17)	0.437
Body mass index ≥25	0.66 (0.59–0.74)	<0.001
Previous MI	1.12 (1.01–1.25)	0.031
History of HF	2.13 (1.88–2.41)	<0.001
NYHA functional class IV	1.17 (0.97–1.41)	0.097
Peripheral vascular disease	1.52 (1.35–1.72)	<0.001
Renal insufficiency	2.99 (2.64–3.38)	<0.001
Hypertension	1.18 (1.04–1.34)	0.008
Diabetes mellitus	1.38 (1.24–1.53)	<0.001
Hyperlipidemia	0.72 (0.65–0.79)	<0.001
Family History of CAD	0.86 (0.76–0.98)	0.026
Smoking	1.16 (1.04–1.29)	0.008
No. of diseased vessels		
1-vessel disease	for reference	
2-vessel disease	1.15 (1.01–1.30)	0.032
3-vessel disease	1.41 (1.22–1.62)	<0.001
Race		
Japanese	for reference	
Asian American	0.84 (0.38–1.89)	0.675
Caucasian	1.56 (1.35–1.79)	<0.001
Black	2.03 (1.62–2.56)	<0.001
Hispanic	1.53 (1.22–1.93)	<0.001

Abbreviation: HR, hazard ratio; CI, confidence interval; NYHA, New York Heart Association; CAD, coronary artery disease.

Multivariate-Adjusted for age ≥65, gender, obesity, history of MI, diabetes, heart failure, NYHA functional class, peripheral vascular disease, renal function, hypertension, hyperlipidemia, family history of CAD, smoking, the number of diseased vessels, and ethnicities. All analyses were stratified by the revascularization procedure.

The risk of long-term mortality was significantly lower among Japanese patients than among other ethnicities except for Asian-Americans (Caucasians: HR, 1.56; 95% CI, 1.35–1.79; p < 0.001; Hispanics: HR, 1.53; 95% CI, 1.22–1.93; p < 0.001; and African-Americans: HR, 2.03; 95% CI, 1.62–2.56; p < 0.001). There was no statistically significant difference in the mortality risk for Asian-Americans when compared with Japanese patients.

## Discussion

Differences have been noted in the long-term outcomes of revascularized patients when comparing patients in Japan and the US. For the patients, from two large-scale registries, who were included in the current analysis, the Japanese patients had significantly better outcomes compared to any ethnic group in the US, except for Asian-Americans, who had outcomes similar to those of the Japanese patients.

Precise reasons remain unclear; however, several explanations can be given for the better cardiovascular outcomes among Japanese patients compared to American patients. First, the prevalence of risk factors might contribute to the different cardiovascular outcomes. In the Japanese population, the risk profiles, (excluding age, smoking, and diabetes mellitus), were consistently better than those of the US population. Moreover, their unique features could overcome any disadvantages in the risk profiles. The Japanese population has one of the highest life expectancies in the world, a lower prevalence of chronic obstructive lung disease despite the higher smoking rate,[3, 13] and a weaker impact of diabetes on cardiovascular outcomes compared to the US population.[14] Second, the characteristics of the coronary artery plaques also vary among ethnic groups. A previous study used intravascular ultrasound to compare the coronary artery plaque morphology between Caucasian and Japanese patients with left main CAD. The study demonstrated that Caucasians had more coronary artery plaque calcification than their Japanese counterparts, even after adjusting for traditional risk factors.[15] Because coronary artery calcification is considered to be a maker of the overall atherosclerotic plaque burden and provides incremental prognostic information,[16] this morphological difference might contribute to the better prognosis for the Japanese population. Recently, a sub-analysis from the Coronary Computed Tomography Angiography Evaluation for Clinical Outcomes: An International Multicenter Registry demonstrated that East-Asian patients with obstructive CAD, as observed using coronary computed tomography angiography, had lower rates of all-cause mortality and nonfatal MI compared to Caucasians and African-Americans during a 2-year follow-up.[17] This result might also indicate a reduced vulnerability of coronary artery plaque development among East-Asian populations. Third, differences in diet, especially the intake of marine-derived n-3 fatty acids, may contribute to the better outcomes in the Japanese population. Compared to the Caucasians and Japanese-Americans in the US, the Japanese have two higher levels of marine-derived n-3 fatty acids, which have anti-atherogenic properties.[18] In addition to n-3 fatty acids, Japanese population consumed higher percentages of total calories from carbohydrates, vegetable protein, eicosapentaenoic acid (EPA), decosahexaenoic acid (DHA), decosapentanoic acid (DPA), compared to the US population.[19] Especially in these components, DHA had an inverse association with intima-medial thickness even after adjusting for other risk factors.[20] Furthermore, on the basis of the studies comparing native Japanese subjects and Japanese-Americans living in the US (mostly Hawai), the strong impact of westernized food habit on unfavorable lipid profiles and progression of atherosclerosis was clarified.[21–23] Despite the genetically identical cohorts, the mean serum cholesterol, and serum triglyceride levels were significantly higher in the Japanese-Americans,[21] the prevalence of type 2 diabetes and death from ischemic heart disease among Japanese-American patients were higher than those among Japanese patients,[22] and IMT was significantly greater in Japanese-Americans than native Japanese.[23] Finally, ethnicity also affects the clinical outcomes. In both clinical registries and trials focused on PCI, the rate of application of optimized medical therapy was not superior to that in the US.[24, 25] This indicates that the better outcomes in Japanese patients are related to genetics or ethnicity rather than to better quality of care.

Previous publications investigating cardiovascular outcome differences, among ethnic groups, have placed a disproportionate emphasis on comparing Caucasians and African-Americans.[7, 26–31] Multiple baseline risk factors and lower accessibility to medical care are generally cited as major factors for the worse cardiovascular outcomes among African-Americans.[31, 32] However, only a few previous PCI outcome studies involving East-Asians have been published, [7, 8, 27] despite the fact that East-Asians have one of the highest increasing rates among various US ethnicities.[33] Thus, further investigation into the prognostic impact of East-Asian ethnicity on cardiovascular outcomes should be undertaken. Our results indicated that the better prognostic value associated with Asian ethnicity was persistent, regardless of the place of patient residence. This is in agreement with a recent study involving the National Cardiovascular Data Registry’s CathPCI Registry, which is linked to Medicare claims data. In that study, Asian patients, revascularized in the US, demonstrated a lower adjusted risk for adverse cardiovascular events than did Caucasian patients.[7]

Our results expand the favorable cardiovascular outcomes associated with Asian ethnicity to post-revascularization patients. The fact that the outcomes of either PCI or CABG revascularizations are different among ethnicities may have an effect on the choice of CAD management strategy for patient of different ethnicity, and may influence the interpretation of trials or studies performed in Western countries when applied to East-Asian populations. As such, the effects of a particular intervention or treatment may differ considerably among different ethnic groups.

### Limitation

Our study has several limitations. First, this study was based on registry data and only included patients who were referred for coronary revascularization. The resulting clinical outcomes of these patients might be quite different depending on any referral bias. Second, generalizability is a major problem since the US dataset was derived from a single institute. In order to demonstrate the generalizability of the US dataset, we compared our US dataset with the National Cardiovascular Data Registry (NCDR), which is the largest national clinical registry program for PCI with more than 1500 centers currently participating across the US (S1 Table).[3] Although the percentages of Hispanics differed significantly (12.2% for THIRDBase vs. 4.9% for NCDR, respectively) between the 2 registries, the background characteristics of the patients appeared identical between the two groups. In order to establish the robustness of this study, it is necessary to utilize a nationwide multicenter dataset for further investigation. Nevertheless, we expect this study to be hypothesis generating, rather than a proof-of-concept. Third, the conclusions were based on the results of a study with a small sample size of Asian Americans in the US dataset. Although the statistical robustness of this conclusion was confirmed, larger registries are needed for further investigation. Forth, statistical adjustment was only made for variables that were collected in both registries, and several variables remained unadjusted. Genetic and pharmacogenomic differences might also contribute to the observed outcome differences, but were beyond the scope of this study. Fifth, technological advances may quickly render our findings out-of-date. One such advance is the advent of drug-eluting stents, which have dramatically reduced the rate of restenosis after stent implantation. However, previous reports have indicated that there is little impact of ethnicity on cardiovascular outcomes after revascularization with different types of stents (bare-metal versus drug-eluting stents).[7] Finally, we are unable to obtain information on the 5% of patients that were lost to follow-up after 2 years in the CREDO-dataset. Although this group might have a slight impact on the result of this study, the conclusion should be statistically robust due to its excellent follow-up rate.

## Conclusion

The adjusted long-term mortality of Japanese patients undergoing revascularization was lower than those for Caucasians, Hispanics, and African-Americans and comparable to that of Asian-American patients in the US.

## Supporting Information

S1 Table Baseline characteristics of each registry. (DOCX)

## References

[1] The Japanese Circulation Society. JCS National Survey on Management of Cardiovascular Diseases: Annual Report. 2011.

[2] Epstein AJ, Polsky D, Yang F, Yang L, Groeneveld PW. Coronary revascularization trends in the United States, 2001–2008. JAMA. 2011;305(17):1769–76. Epub 2011/05/05. 10.1001/jama.2011.551 21540420 PMC3164857

[3] Dehmer GJ, Weaver D, Roe MT, Milford-Beland S, Fitzgerald S, Hermann A, et al A contemporary view of diagnostic cardiac catheterization and percutaneous coronary intervention in the United States: a report from the CathPCI Registry of the National Cardiovascular Data Registry, 2010 through June 2011. J Am coll Cardiol. 2012;60(20):2017–31. Epub 2012/10/23. 10.1016/j.jacc.2012.08.966 . 23083784

[4] Amano J, Kuwano H, Yokomise H. Thoracic and cardiovascular surgery in Japan during 2011: Annual report by The Japanese Association for Thoracic Surgery. Gen Thorac Cardiovasc Surg. 2013;61(10):578–607. 10.1007/s11748-013-0289-2 . 23990117

[5] Palaniappan L, Wang Y, Fortmann SP. Coronary heart disease mortality for six ethnic groups in California, 1990–2000. Ann Epidemiol. 2004;14(7):499–506. Epub 2004/08/18. 10.1016/j.annepidem.2003.12.001 . 15310526

[6] Shaw LJ, Shaw RE, Merz CN, Brindis RG, Klein LW, Nallamothu B, et al Impact of ethnicity and gender differences on angiographic coronary artery disease prevalence and in-hospital mortality in the American College of Cardiology-National Cardiovascular Data Registry. Circulation. 2008;117(14):1787–801. Epub 2008/04/02. 10.1161/CIRCULATIONAHA.107.726562 . 18378615

[7] Kumar RS, Douglas PS, Peterson ED, Anstrom KJ, Dai D, Brennan JM, et al Effect of race and ethnicity on outcomes with drug-eluting and bare metal stents: results in 423 965 patients in the linked National Cardiovascular Data Registry and centers for Medicare & Medicaid services payer databases. Circulation. 2013;127(13):1395–403. Epub 2013/04/03. 10.1161/CIRCULATIONAHA.113.001437 . 23547179

[8] Kohsaka S, Kimura T, Goto M, Lee VV, Elayda M, Furukawa Y, et al Difference in patient profiles and outcomes in Japanese versus American patients undergoing coronary revascularization (collaborative study by CREDO-Kyoto and the Texas Heart Institute Research Database). Am J Cardiol. 2010;105(12):1698–704. Epub 2010/06/12. 10.1016/j.amjcard.2010.01.349 . 20538117

[9] Mehta RH, Parsons L, Peterson ED, National Registry of Myocardial Infarction I. Comparison of bleeding and in-hospital mortality in Asian-Americans versus Caucasian-Americans with ST-elevation myocardial infarction receiving reperfusion therapy. Am J Cardiol. 2012;109(7):925–31. 10.1016/j.amjcard.2011.11.019 . 22221945

[10] Xu W, Holmes DN, Becker RC, Roe MT, Peterson ED, Wang TY. Comparison of long-term outcomes between older Asian and white patients with non-ST-segment elevation myocardial infarction: findings from CRUSADE-CMS database. Am Heart J. 2013;166(6):1050–5. 10.1016/j.ahj.2013.10.001 . 24268220

[11] Wang TY, Chen AY, Roe MT, Alexander KP, Newby LK, Smith SC Jr., et al Comparison of baseline characteristics, treatment patterns, and in-hospital outcomes of Asian versus non-Asian white Americans with non-ST-segment elevation acute coronary syndromes from the CRUSADE quality improvement initiative. Am J Cardiol. 2007;100(3):391–6. 10.1016/j.amjcard.2007.03.035 . 17659915

[12] Kohsaka S, Goto M, Virani S, Lee VV, Aoki N, Elayda MA, et al Long-term clinical outcome of coronary artery stenting or coronary artery bypass grafting in patients with multiple-vessel disease. J Thorac Cardiovasc Surg. 2008;136(2):500–6. Epub 2008/08/12. 10.1016/j.jtcvs.2007.11.050 . 18692664

[13] Nishiyama K, Morimoto T, Furukawa Y, Nakagawa Y, Ehara N, Taniguchi R, et al Chronic obstructive pulmonary disease—an independent risk factor for long-term cardiac and cardiovascular mortality in patients with ischemic heart disease. Int J Cardiol. 2010;143(2):178–83. 10.1016/j.ijcard.2009.02.010 . 19368979

[14] Kohsaka S, Goto M, Nagai T, Lee VV, Elayda M, Furukawa Y, et al Impact of diabetes among revascularized patients in Japan and the U.S. Diabetes care. 2012;35(3):654–9. Epub 2012/02/04. 10.2337/dc11-1547 22301120 PMC3322729

[15] Rusinova RP, Mintz GS, Choi SY, Araki H, Hakim D, Sanidas E, et al Intravascular ultrasound comparison of left main coronary artery disease between white and Asian patients. Am J Cardiol. 2013;111(7):979–84. Epub 2013/01/24. 10.1016/j.amjcard.2012.12.014 . 23340034

[16] Kim SW, Mintz GS, Escolar E, Ohlmann P, Pregowski J, Tyczynski P, et al The impact of cardiovascular risk factors on subclinical left main coronary artery disease: an intravascular ultrasound study. Am Heart J. 2006;152(4):693 e7–12. Epub 2006/09/26. 10.1016/j.ahj.2006.07.012 . 16996839

[17] Hulten E, Villines TC, Cheezum MK, Berman DS, Dunning A, Achenbach S, et al Usefulness of coronary computed tomography angiography to predict mortality and myocardial infarction among Caucasian, African and East Asian ethnicities (from the CONFIRM [Coronary CT Angiography Evaluation for Clinical Outcomes: An International Multicenter] Registry). Am J Cardiol. 2013;111(4):479–85. Epub 2012/12/06. 10.1016/j.amjcard.2012.10.028 . 23211358

[18] Sekikawa A, Curb JD, Ueshima H, El-Saed A, Kadowaki T, Abbott RD, et al Marine-derived n-3 fatty acids and atherosclerosis in Japanese, Japanese-American, and white men: a cross-sectional study. J Am Coll Cardiol. 2008;52(6):417–24. 10.1016/j.jacc.2008.03.047 18672160 PMC2736602

[19] Nakamura Y, Ueshima H, Okuda N, Higashiyama A, Kita Y, Kadowaki T, et al Relation of dietary and other lifestyle traits to difference in serum adiponectin concentration of Japanese in Japan and Hawaii: the INTERLIPID Study. Am J Clin Nutr. 2008;88(2):424–30. . 18689379 10.1093/ajcn/88.2.424 PMC6660152

[20] Sekikawa A, Kadowaki T, El-Saed A, Okamura T, Sutton-Tyrrell K, Nakamura Y, et al Differential association of docosahexaenoic and eicosapentaenoic acids with carotid intima-media thickness. Stroke. 2011;42(9):2538–43. 10.1161/STROKEAHA.110.613042 21757663 PMC3164236

[21] Egusa G, Murakami F, Ito C, Matsumoto Y, Kado S, Okamura M, et al Westernized food habits and concentrations of serum lipids in the Japanese. Atherosclerosis. 1993;100(2):249–55. . 8357357 10.1016/0021-9150(93)90211-c

[22] Nakanishi S, Okubo M, Yoneda M, Jitsuiki K, Yamane K, Kohno N. A comparison between Japanese-Americans living in Hawaii and Los Angeles and native Japanese: the impact of lifestyle westernization on diabetes mellitus. Biomed Pharmacother. 2004;58(10):571–7. 10.1016/j.biopha.2004.10.001 . 15589065

[23] Watanabe H, Yamane K, Fujikawa R, Okubo M, Egusa G, Kohno N. Westernization of lifestyle markedly increases carotid intima-media wall thickness (IMT) in Japanese people. Atherosclerosis. 2003;166(1):67–72. . 12482552 10.1016/s0021-9150(02)00304-0

[24] Nishigaki K, Yamazaki T, Kitabatake A, Yamaguchi T, Kanmatsuse K, Kodama I, et al Percutaneous coronary intervention plus medical therapy reduces the incidence of acute coronary syndrome more effectively than initial medical therapy only among patients with low-risk coronary artery disease a randomized, comparative, multicenter study. JACC Cardiovasc Interv. 2008;1(5):469–79. 10.1016/j.jcin.2008.08.002 . 19463347

[25] Endo A, Kohsaka S, Miyata H, Kawamura A, Noma S, Suzuki M, et al Disparity in the application of guideline-based medical therapy after percutaneous coronary intervention: analysis from the Japanese prospective multicenter registry. Am J Cardiovasc Drugs. 2013;13(2):103–12. 10.1007/s40256-013-0021-8 . 23585142

[26] Thomas KL, Honeycutt E, Shaw LK, Peterson ED. Racial differences in long-term survival among patients with coronary artery disease. Am Heart J. 2010;160(4):744–51. Epub 2010/10/12. 10.1016/j.ahj.2010.06.014 . 20934570

[27] Slater J, Selzer F, Dorbala S, Tormey D, Vlachos HA, Wilensky RL, et al Ethnic differences in the presentation, treatment strategy, and outcomes of percutaneous coronary intervention (a report from the National Heart, Lung, and Blood Institute Dynamic Registry). Am J Cardiol. 2003;92(7):773–8. Epub 2003/10/01. . 14516874 10.1016/s0002-9149(03)00881-6

[28] Pradhan J, Schreiber TL, Niraj A, Veeranna V, Ramesh K, Saigh L, et al Comparison of five-year outcome in African Americans versus Caucasians following percutaneous coronary intervention. Catheter Cardiovasc Interv. 2008;72(1):36–44. Epub 2008/04/03. 10.1002/ccd.21556 . 18383170

[29] Berger JS, Sanborn TA, Sherman W, Brown DL. Comparison of three-year outcomes in blacks versus whites with coronary heart disease following percutaneous coronary intervention. Am J Cardiol. 2004;94(5):647–9. Epub 2004/09/03. 10.1016/j.amjcard.2004.05.033 . 15342300

[30] Marks DS, Mensah GA, Kennard ED, Detre K, Holmes DR Jr. Race, baseline characteristics, and clinical outcomes after coronary intervention: The New Approaches in Coronary Interventions (NACI) registry. Am Heart J. 2000;140(1):162–9. Epub 2000/06/30. 10.1067/mhj.2000.106645 . 10874280

[31] Khambatta S, Seth M, Rosman HS, Share D, Aronow HD, Moscucci M, et al The association between patient race, treatment, and outcomes of patients undergoing contemporary percutaneous coronary intervention: insights from the Blue Cross Blue Shield of Michigan Cardiovascular Consortium (BMC2). Am Heart J. 2013;165(6):893–901 e2 Epub 2013/05/28. 10.1016/j.ahj.2013.02.030 . 23708159

[32] Peterson ED, Shaw LK, DeLong ER, Pryor DB, Califf RM, Mark DB. Racial variation in the use of coronary-revascularization procedures. Are the differences real? Do they matter? N Engl J Med. 1997;336(7):480–6. Epub 1997/02/13. 10.1056/NEJM199702133360706 . 9017942

[33] US Department of Health and Human Services Centers for Disease Ccontrol and Prevention, National Center for Health Statistics. Health, United States 2005 with Chartbook on Trends in the Health of Americans. Hyattsville, Maryland: 2005.

