# Supplementary material for: Hypothesis of Long-Term Outcome after Coronary Revascularization in Japanese Patients Compared to Multiethnic Groups in the US
Source: PLoS One. 2015 May 29;10(5):e0128252. doi: 10.1371/journal.pone.0128252 (PMC4449105; doi:10.1371/journal.pone.0128252)
Supplement: S1 Table — (DOCX) [file pone.0128252.s001.docx]

| S1 Table. Baseline characteristics of each registry | | | |
| --- | --- | --- | --- |
|  |  | THIRDBase | NCDR |
|  |  | N=6717 | N=941248 |
| Age (>65 years) (%) | | 43.4 | 51 |
| Gender (Female) (%) | | 30.3 | 32.6 |
| Ethnicity | |  |  |
|  | Caucasians (%) | 77 | 88.3 |
|  | African-American (%) | 9.6 | 8.1 |
|  | Hispanic (%) | 12.2 | 4.9 |
|  | Asian-American (%) | 1.2 | 2.1 |
|  | Others (%) | 0 | 0.7 |
| Obesity (BMI <25 kg/m2) (%) | | 75.2 | 79.3 |
| Previous MI (%) | | 34.1 | 30 |
| Previous HF (%) | | 14.3 | 11.8 |
| Peripheral vascular disease (%) | | 14.9 | 12.5 |
| Renal insufficiency (%) | | 11.5 | 27.2 |
| Hypertension (%) | | 73.9 | 82 |
| Diabetes mellitus (%) | | 30.8 | 36.2 |
| Dyslipidemia (%) | | 60.9 | 80 |
| Abbreviations: NCDR, National Cardiovascular Data Registry; BMI, body mass index; MI, myocardial infarction; HF, heart failure. | | | |
|  |  |  |  |
